# Association of epicardial adipose tissue with markers of cardiac remodelling and clinical outcomes in asymptomatic aortic stenosis

**DOI:** 10.1136/openhrt-2025-003804

**Published:** 2026-02-09

**Authors:** Sarah L Ayton, Saadia Aslam, Abhishek Dattani, Jian L Yeo, Gaurav S Gulsin, Piotr J Slomka, Damini Dey, Gerry P McCann, Anvesha Singh

**Affiliations:** 1 Department of Cardiovascular Sciences, Leicester British Heart Foundation Centre of Research Excellence and Leicester National Institute for Health and Care Research Biomedical Research Centre, University of Leicester, Leicester, UK; 2 Biomedical Imaging Research Institute, Cedars-Sinai Medical Center, Los Angeles, California, USA

**Keywords:** aortic valve stenosis, magnetic resonance imaging, multidetector computed tomography, obesity

## Abstract

**Background:**

Previous studies have suggested an adverse role of epicardial adipose tissue (EAT) in aortic stenosis (AS), potentially mediated by direct effects on the myocardium. This study aimed to assess whether EAT volume and density are associated with cardiac remodelling and clinical outcomes in a well-phenotyped cohort of patients with initially asymptomatic AS.

**Methods:**

In this post hoc exploratory analysis of a multicentre, prospective longitudinal study, asymptomatic patients with moderate-to-severe AS (n=136; age 68.6 (60.1, 75.3) years, 27% female) and control participants (n=39; age 59.0 (54.0, 67.0) years, 38% female) underwent echocardiography, cardiac CT and MRI. EAT volume and mean CT attenuation were measured from non-contrast cardiac CT using an automated deep learning software. The primary outcome was symptoms necessitating valve replacement, cardiovascular death or major adverse cardiac event.

**Results:**

Participants with AS had significantly higher indexed EAT volumes (56.5 vs 38.8 cm^3^/m^2^, p<0.001) and lower EAT attenuation (−76.3 vs −69.9 Hounsfield units, p<0.001). Both measures were correlated with markers of AS severity. Indexed EAT volume was not associated with cardiac remodelling in AS after correcting for confounding variables; however, a higher mean EAT CT attenuation was associated with higher indexed left ventricular mass. There were 42 (31%) primary outcome events over a median of 370 days. Lower indexed EAT volume and higher mean CT attenuation were associated with the outcome, independent of age and sex. Indexed EAT volume remained negatively associated after further adjustment for AS severity.

**Conclusion:**

Indexed EAT volume was higher in patients with AS, but not associated with MRI markers of cardiac remodelling. A higher indexed EAT volume was independently associated with a lower occurrence of symptoms and cardiovascular events. Further studies are needed to corroborate these findings and whether EAT has a potentially protective role in symptom progression in AS.

**Trial registration numbers:**

NCT03518645, NCT03132129.

**WHAT IS ALREADY KNOWN ON THIS TOPIC**

- Previous studies have reported higher epicardial adipose tissue (EAT) volumes in patients with aortic stenosis (AS) and EAT has been shown to be associated with adverse events in some studies.

**WHAT THIS STUDY ADDS**

- In this cohort of asymptomatic patients with moderate-to-severe AS, indexed EAT volume was higher than controls, but not independently associated with MRI markers of left ventricular remodelling.
- Patients with a higher indexed EAT volume were at lower risk of the occurrence of typical AS symptoms necessitating valve replacement and cardiovascular events, and this association remained after adjustment for AS severity.

**HOW THIS STUDY MIGHT AFFECT RESEARCH, PRACTICE OR POLICY**

- This suggests a possible protective role of EAT in asymptomatic AS and further research into this is required.

## Introduction

 Aortic stenosis (AS) is characterised by progressive thickening and calcification of the aortic valve. The obstructive valve results in left ventricular (LV) hypertrophy, which is initially adaptive to maintain cardiac output, but becomes maladaptive over time, with onset of fibrosis, microvascular dysfunction and diastolic and systolic dysfunction.^1^ In the high-income world, it is the most common valvular heart disease and its prevalence is increasing as the population ages.^2^ Surgical or transcatheter valve replacement is the only available treatment and improves prognosis once symptoms develop.^3^ The identification of factors associated with disease progression may allow for better risk-stratification of patients for prompt intervention before irreversible cardiac remodelling occurs, as well as identification of potential therapeutic targets.

Obesity is known to be a risk factor for several cardiac diseases, including atherosclerotic cardiovascular disease and heart failure. Both increased body mass index (BMI) and waist circumference have been found to be associated with an increased incidence of AS in several large studies.^4 5^ However, studies have found differing effects of obesity relating to the progression of AS,^6 7^ with some suggestion of an ‘obesity paradox’, whereby increased body weight offers a protective effect, including more favourable outcomes after transcatheter aortic valve intervention.^8^

Increased deposition of visceral adipose tissue, contributing to a chronic pro-inflammatory state, appears central to the adverse consequences of obesity and metabolic disease. Epicardial adipose tissue (EAT) is a visceral adipose tissue deposit of interest within cardiovascular disease, due to its proximity to the heart. It can exert direct effects on the myocardium, as there is no fascial barrier between them.^9^ Patients with AS have been shown to have increased EAT thickness compared with healthy controls,^10 11^ with increased production of inflammatory mediators by larger EAT deposits.^11^ Release of inflammatory and pro-calcific factors into the circulation may directly impact the progression of aortic valve disease and substances from EAT directly secreted into the myocardium may influence LV remodelling.^12^ Two previous studies have found associations with increased EAT volume and adverse events, including mortality, in patients with AS.^13 14^

This study aimed to assess whether the volume and density of EAT, measured using CT, was associated with measures of cardiac remodelling and the onset of symptoms or adverse cardiovascular events, in initially asymptomatic patients with moderate-to-severe AS.

## Materials and methods

### Study population

This is a post hoc exploratory analysis of the ‘PRognostic Importance of MIcrovascular Dysfunction in asymptomatic patients with AS’ (PRIMID-AS) study (NCT01658345). This was a multicentre, prospective, observational study, which recruited participants with asymptomatic moderate-to-severe AS. It was conducted in the UK between April 2012 and November 2014 to assess the prognostic role of multiparametric cardiac MRI, in particular myocardial perfusion reserve (MPR).^15^ Individuals aged 18–85 years with asymptomatic moderate to severe AS were included. The full inclusion and exclusion criteria have been previously published.^15^ The sample size for the PRIMID-AS study was determined based on 80% power to show MPR had superior accuracy in predicting symptom onset compared with exercise testing, as this was the primary aim of the original study. This analysis is a post hoc analysis of the main study. Participants with CT scans that were analysable for EAT measurements are included in this report.

To act as a comparator group and define normal EAT values in our regional population, control participants with no history, signs or symptoms of cardiovascular disease (symptomatic coronary, peripheral or cerebrovascular disease, valvular heart disease, arrhythmias or heart failure) recruited into the ‘Prevalence and Determinants of Subclinical Cardiovascular Dysfunction in Adults with Type 2 Diabetes’ study (NCT03132129) are included in this report.^16^ Use of statin therapy and controlled hypertension were not exclusion criteria in this control cohort.

Patient and public involvement (PPI) members were involved during the conduct of the original PRIMID-AS research study, with details shared with the local cardiovascular PPI group and feedback incorporated. A lay member was also part of the study steering committee.

### Study procedures

All participants underwent comprehensive cardiovascular imaging at baseline, including transthoracic echocardiography, non-contrast cardiac CT and multiparametric cardiac MRI. Demographic and anthropometric data were collected. Body surface area was calculated using the Mosteller formula.^17^

### Image acquisition and analysis

#### Non-contrast CT

Non-contrast CT scans were acquired on a multidetector CT scanner with prospective electrocardiographic gating triggering at 70% of the R-R interval and a tube voltage of 120 kVp. Raw data were reconstructed at a slice thickness of 3.0 mm.

**Figure 1:**
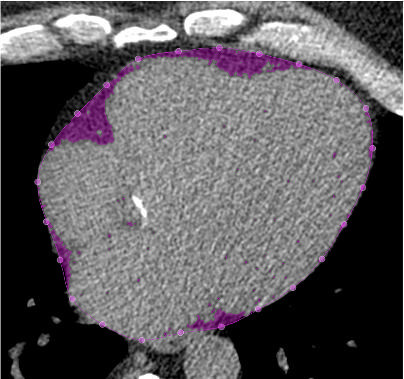
An example CT slice from a participant with aortic stenosis. Pericardial contours are computed using a deep learning model every three slices, then interpolated between slices. Inside the contour, pixels with Hounsfield units of −190 to −30 are seen highlighted in purple, identifying epicardial adipose tissue (EAT). EAT volume and mean CT attenuation are then calculated.

EAT volume and mean CT attenuation (which represents tissue density) were measured using a fully automated deep learning software (QFat, V.2.0; Cedars-Sinai Medical Center, Los Angeles, California),^18^ as previously described.^16^ Figure 1 shows an example CT slice with contours. Adipose tissue was defined using the Hounsfield unit range of −190 to −30.^18^ Minimal adjustments were made to contours. EAT volume was indexed to body surface area.

#### Cardiac MRI

Cardiac MRI was performed using 3 T platforms and included standard balanced steady-state free precession (bSSFP) cine imaging in long-axis and short-axis planes, rest and adenosine stress first-pass perfusion and late gadolinium enhancement imaging.^15^

Analyses for LV mass, volumes and strain were performed using cvi42 (V.5.10.1, Circle Cardiovascular Imaging, Calgary, Canada), as described elsewhere.^19^ For ease of interpretation, strain measurements are presented as positive values, with a lower value indicating worse strain. In participants with AS, rest and stress myocardial blood flow were calculated using model independent deconvolution, with contouring done using Q-mass V.7.1 (Medis, Leiden, The Netherlands) and in control participants, inline automated reconstruction and postprocessing was used.^20^ MPR was calculated as the ratio of stress and rest myocardial blood flow. The presence and distribution of late gadolinium enhancement was agreed by two observers.

#### Echocardiography

A comprehensive transthoracic echocardiogram was performed in participants with AS, according to international guidelines, by British Society of Echocardiography accredited cardiac physiologists.

### Follow-up of participants with aortic stenosis

The primary outcome was a composite of typical AS symptoms necessitating referral for aortic valve replacement (AVR), cardiovascular death or a major adverse cardiac event (hospitalisation with heart failure, chest pain, syncope, arrhythmia). Participants were followed up every 6 months for a minimum of 12 months or until the end point was reached. An independent committee adjudicated events for inclusion as a primary outcome.

### Statistical analysis

Data were assessed for symmetrical distribution and normality using the Shapiro-Wilk test, histograms and Q-Q plots. Continuous, symmetrically distributed data are presented as mean (SD) and non-normally distributed data as median (IQR). Categorical data are presented as frequency (percentage). The two-sample t-test and Wilcoxon rank sum test were used, as appropriate, after assessment of normality and homogeneity of variance, to compare imaging variables between AS and control cohorts. The correlation between indexed EAT volume and mean CT attenuation was assessed using Pearson’s correlation co-efficient.

In participants with AS, the correlations between CT-derived EAT measures and measures of AS severity from echocardiography were assessed using scatter plots and Pearson’s correlation co-efficient. The associations between EAT and MRI measures of LV remodelling were assessed using scatter plots and univariable linear regression analysis. To further assess these associations, initial multivariable regression models with variables known to be associated with LV remodelling in AS were performed for each MRI measure of LV remodelling. This model included age, gender, BMI, systolic blood pressure, diabetic status and indexed aortic valve area (AVA). Indexed EAT volume and mean CT attenuation were then added to this model separately to assess their independent association with each MRI marker. All linear regression analyses were assessed for the underlying assumptions of normality, homogeneity of variance of residuals and linearity for quantitative predictors, as well as for multicollinearity in multivariable analyses.

The association between EAT measurements and the primary outcome (time to event) was assessed using Cox regression analyses. Kaplan-Meier curves were constructed with the log-rank test and the optimal cut-off for each EAT measurement was obtained using receiver operating characteristic curves and the Youden index. The start and end time points were defined, respectively, as recruitment into the study and either the time of the end point occurring or the final follow-up time point, which was at least 12 months. Multivariable Cox regression analyses were performed with age and gender (which are both known to be associated with the outcome) and each EAT measurement as a continuous variable (‘model 1’). To assess whether any associations were independent of BMI and diabetic status, which are known to be associated with increased EAT volume,^16^ as well as indexed AVA, as severity of AS is known to be associated with the outcome, these measures were added to model 1 in a further multiple Cox regression analysis (‘model 2’). The assumptions of proportional hazards and linearity for quantitative predictors were assessed.

Statistical analyses were performed using the R environment for statistical computing.^21^ A p value <0.05 was considered statistically significant.

## Results

**Figure 2:**
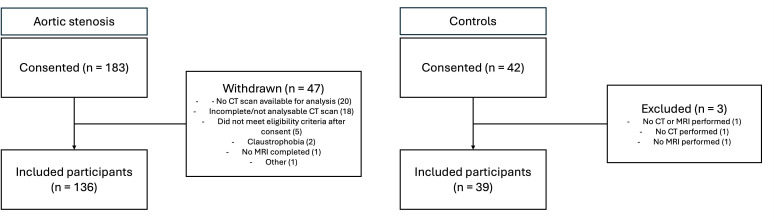
Participants with aortic stenosis and controls are included in this report.

Of the 174 participants included in the PRIMID-AS study,^15^ 136 individuals had analysable CT scans available (figure 2). Thirty-nine control participants were included. Participants with AS were older, with a higher BMI and systolic blood pressure compared with controls (table 1). Additionally, there was a higher prevalence of hyperlipidaemia. One-third of participants with AS had concomitant coronary artery disease and 13% had type 2 diabetes. 60% of participants with AS were taking a statin, compared with 21% of the control participants.

**Table 1:** Demographics and anthropometric measurements

	Aortic stenosis (n=136)	Controls (n=39)
Age (years)	68.6 (60.1, 75.3)	59.0 (54.0, 67.0)
Sex (female)	37 (27%)	15 (38%)
BMI (kg/m^2^)	28.1±3.9	26.2±3.5
Weight (kg)	81.1±13.8	78.0±16.3
Systolic blood pressure (mm Hg)	143.8 (130.3, 161.8)	122.0 (112.0, 133.0)
Diastolic blood pressure (mm Hg)	76.5±10.9	74.9±7.9
Smoking history		
Current smoker	11 (8.1%)	0
Ex-smoker	57 (42%)	17 (44%)
Never smoked	68 (50%)	22 (56%)
Hypertension	69 (51%)	6 (15%)
Hyperlipidaemia	71 (58%)	7 (18%)
Obesity	43 (32%)	9 (23%)
Diabetes	17 (13%)	0
Coronary artery disease	46 (34%)	0
History of cardiac arrhythmia	10 (7.4%)	0
Medication use		
ACE inhibitors	39 (29%)	4 (10%)
Beta-blockers	41 (30%)	0
Calcium channel blockers	37 (27%)	1 (2.6%)
Diuretics	36 (26%)	1 (2.6%)
Angiotensin receptor blockers	23 (17%)	0
Alpha-blockers	5 (3.7%)	0
Statins	81 (60%)	8 (21%)
Ezetimibe	4 (2.9%)	0
Fibrates	4 (2.9%)	0

Mean±SD, median (IQR) or n (%).

BMI, body mass index.

In participants with AS, median AVA was 1.1 (0.9, 1.4) cm^2^, mean pressure gradient was 32.3 (26.0, 40.5) mm Hg and median peak velocity 3.7 (3.5, 4.2) m/s, with 96 (71%) of participants meeting one criterion for severe AS (online supplemental table 1). Cardiac MRI results are presented in table 2.

**Table 2:** CT and MRI results for participants with AS and controls

	Aortic stenosis (n=136)	Controls (n=39)	P value
EAT volume (cm^3^)	110.2 (67.9, 135.1)	76.2 (46.7, 102.8)	<0.001
Indexed EAT volume (cm^3^/m^2^)	56.5 (36.6, 71.2)	38.8 (29.1, 52.1)	<0.001
Mean EAT CT attenuation (HU)	−76.3±5.6	−69.9±5.4	<0.001
Aortic valve calcium score	1773.5 (1117.0, 3138.0)	–	–
Coronary calcium score	277.0 (1.4, 934.0)	4.0 (0.0, 149.0)	<0.001
LVMi (g/m^2^)	76.4 (66.5, 88.7)	61.4 (55.5, 70.9)	<0.001
LVM/V	1.0 (0.9, 1.2)	0.8 (0.7, 0.9)	<0.001
LVEDVi (mL/m^2^)	74.9 (65.4, 85.2)	80.5 (68.7, 90.6)	0.062
LVESVi (mL/m^2^)	21.2 (16.9, 29.4)	26.6 (21.9, 31.1)	0.001
LVSVi (mL/m^2^)	53±11.7	52.4±9.1	0.7
LVEF (%)	70.3±7.5	65.8±6.5	<0.001
GLS (%)	14.8±2.6	17.2±2.2	<0.001
Longitudinal PEDSR (1/s)	0.5±0.2	0.7±0.2	<0.001
GCS (%)	18.8±2.8	19.1±2.3	0.5
Circumferential PEDSR (1/s)	0.7±0.2	1.0±0.2	<0.001
Global MPR	2.2 (1.7, 2.7)	3.2 (2.6, 3.5)	<0.001
LGE present (n, %)			
None or insertion point only	76 (56%)	32 (82%)	
Significant	60 (44%)	7 (18%)	

Mean±SD, median (IQR) or n (%).

AS, aortic stenosis; EAT, epicardial adipose tissue; GCS, global circumferential strain; GLS, global longitudinal strain; HU, Hounsfield units; LGE, late gadolinium enhancement; LVEDVi, left ventricular end diastolic volume indexed to body surface area; LVEF, left ventricular ejection fraction; LVESVi, left ventricular end systolic volume indexed to body surface; LVMi, left ventricular mass indexed to body surface area; LVM/V, left ventricular mass to volume ratio; LVSVI, left ventricular stroke volume indexed to body surface; MPR, myocardial perfusion reserve; PEDSR, peak early diastolic strain rate.

### Epicardial adipose tissue measurements

**Figure 3:**
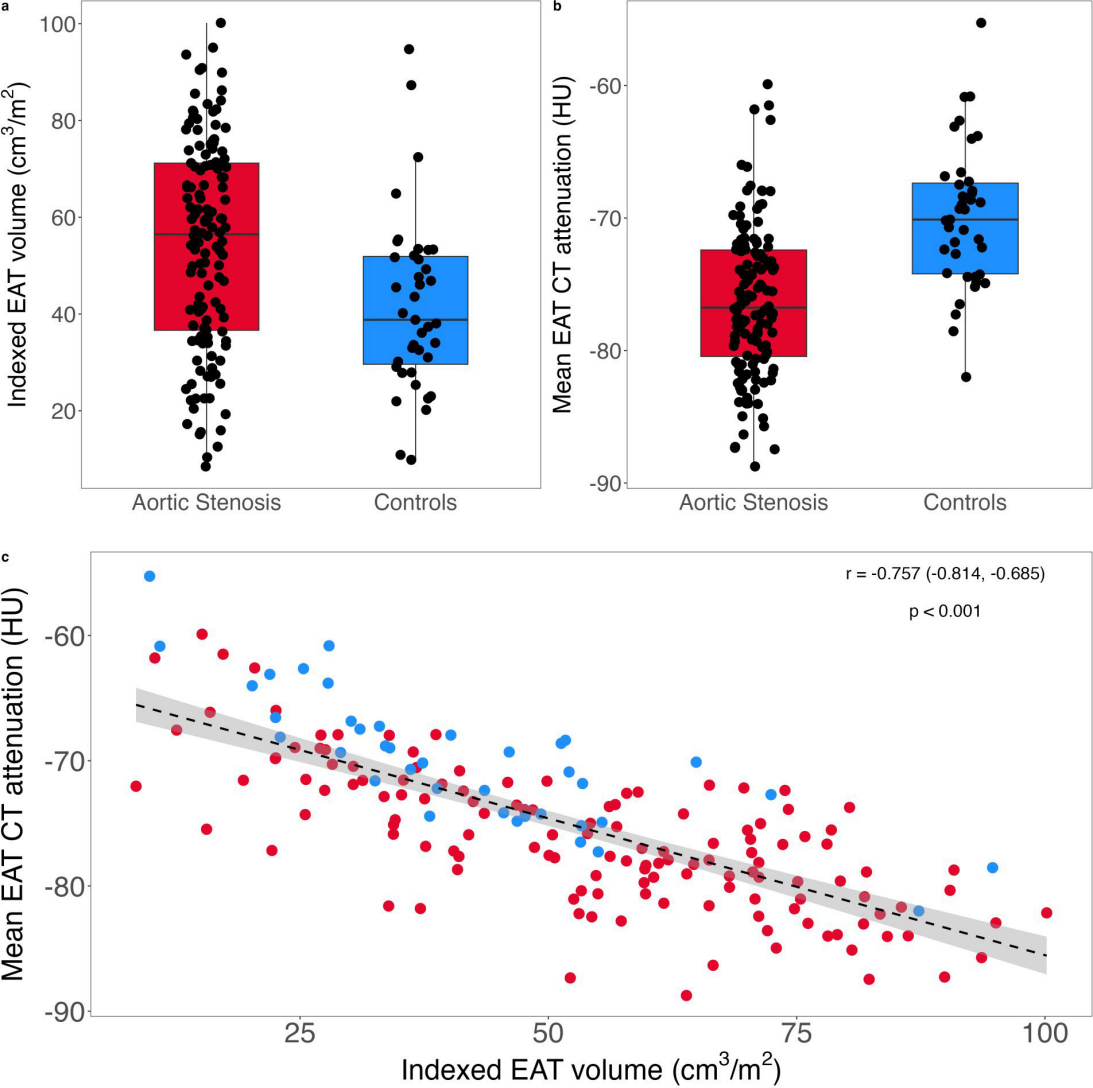
Box plots showing (a) indexed total epicardial adipose tissue (EAT) volume and (b) mean EAT CT attenuation in participants with aortic stenosis and controls (outliers defined as more or less than 1.5 times the IQR), (**c**) scatter plot of the correlation between indexed EAT volume and mean EAT attenuation, with regression line and grey shaded area demonstrating the 95% confidence band. HU, Hounsfield units.

Indexed EAT volume was significantly higher in participants with AS compared with controls, with a lower EAT mean CT attenuation (table 2, figure 3). This was independent of age, which differed between the two cohorts. Indexed EAT volume was negatively correlated with mean CT attenuation (figure 3; r=−0.757, p<0.001).

### Association of EAT with measures of aortic stenosis severity

Increased indexed EAT volume was negatively, but weakly correlated with mean pressure gradient (r=−0.203, p=0.018) and peak aortic jet velocity (r=−0.212, p=0.013) and mean EAT CT attenuation was positively correlated with these measures (r=0.351, p≤0.001 and r=0.353, p≤0.001, respectively; online supplemental figure 1). Neither measure was significantly correlated with indexed AVA (r=0.164, p=0.058 for indexed EAT volume and r=−0.0018, p=0.832 for mean EAT CT attenuation).

### Association of EAT with cardiac MRI parameters

**Figure 4:**
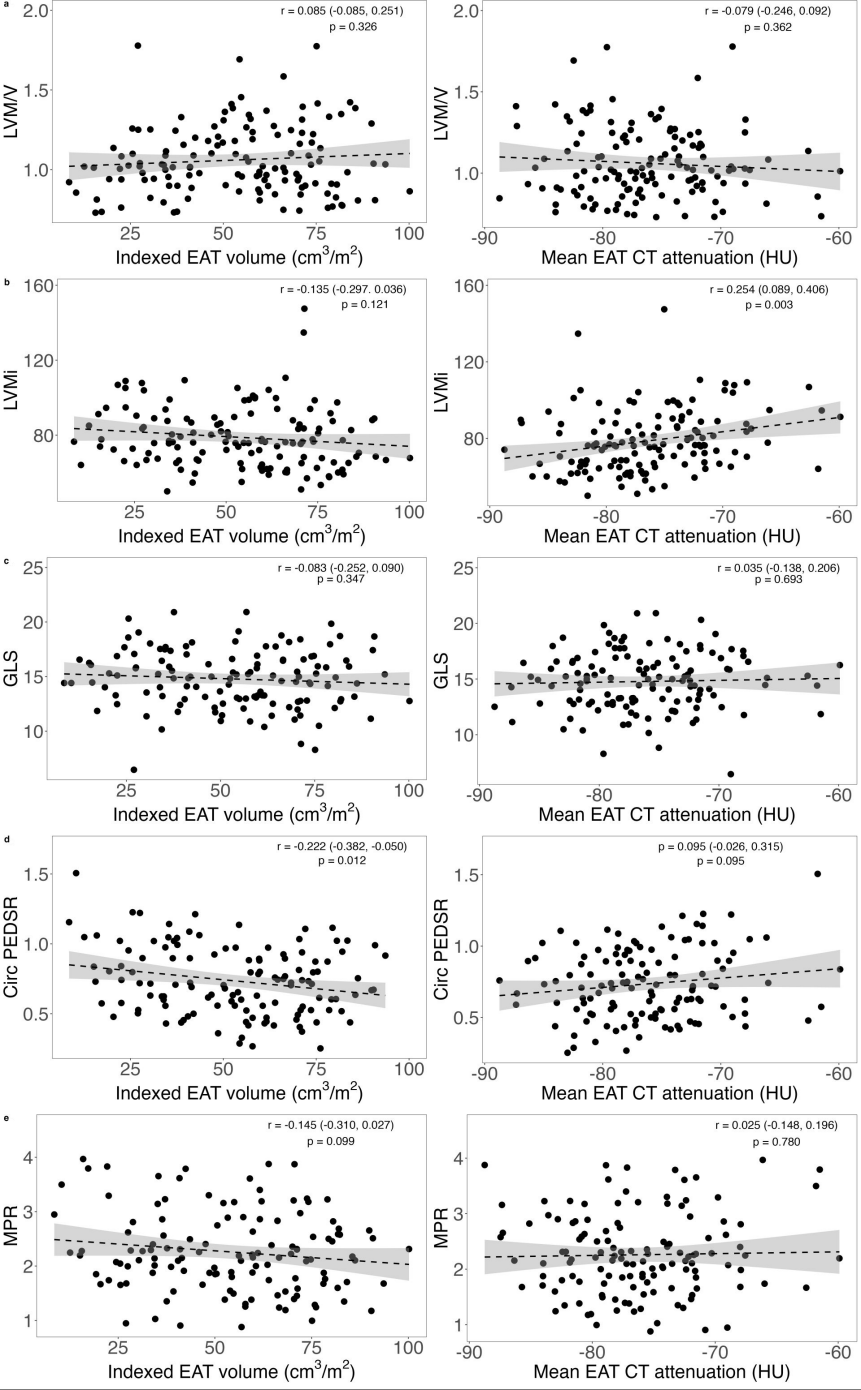
The association between indexed epicardial adipose tissue (EAT) volume and CT mean attenuation and MRI measures of cardiac remodelling, subclinical dysfunction and myocardial perfusion in participants with aortic stenosis (grey shaded area demonstrating the 95% confidence band). GLS, global longitudinal strain; HU, Hounsfield units; LVM/V, left ventricular mass/volume ratio; LVMi, left ventricular mass indexed to body surface area; MPR, myocardial perfusion reserve; PEDSR, peak early diastolic strain rate.

In the AS cohort, increased indexed EAT volume was associated with a lower circumferential PEDSR (adjusted r^2^=−0.042, p=0.012; figure 4) and higher (less negative) mean EAT CT attenuation was associated with a higher indexed LV mass on univariable analysis (adjusted r^2^=0.058, p=0.003; figure 4). When adjusted for age, gender, BMI, systolic blood pressure, diabetic status and indexed AVA, only mean EAT CT attenuation remained associated with indexed LV mass (online supplemental table 2). There was no statistically significant association demonstrated for MPR with either measure of EAT.

### Association of EAT with outcomes in AS

During a median follow-up of 370 days (IQR 311–473 days), the primary outcome occurred in 42 (31%) participants with AS, 40 of whom developed symptoms necessitating referral for AVR. In Cox regression analysis, increased indexed EAT volume and lower mean CT attenuation were associated with lower rates of events, when adjusted for age and sex (table 3; ‘model 1’). Indexed EAT volume remained associated in the same direction with the outcome with additional adjustment for BMI, diabetic status and indexed AVA (‘model 2’); however, mean EAT attenuation was no longer significant. Results were similar when different measures of AS severity were used in the models.

**Table 3:** Cox proportional hazards model for outcomes in aortic stenosis

	Model 1			Model 2		
	HR	95% CI	P value	HR	95% CI	P value
**Models to assess indexed total EAT volume**						
Indexed total EAT volume	0.974	0.958 to 0.989	0.001	0.978	0.961 to 0.995	0.010
Age (per 5-year increase)	1.20	1.05 to 1.37	0.006	1.15	1.01 to 1.31	0.039
Gender (male)	0.521	0.277 to 0.979	0.043	0.525	0.275 to 0.999	0.050
BMI				0.982	0.909 to 1.06	0.639
Diabetes				1.39	0.599 to 3.21	0.445
Indexed AVA				0.018	0.001 to 0.244	0.003
**Models to assess EAT mean attenuation**						
EAT mean attenuation	1.08	1.02 to 1.15	0.012	1.07	0.999 to 1.14	0.054
Age (per 5-year increase)	1.16	1.02 to 1.33	0.028	1.11	0.972 to 1.26	0.124
Gender (male)	0.509	0.270 to 0.960	0.037	0.531	0.279 to 1.01	0.054
BMI				0.995	0.915 to 1.08	0.905
Diabetes				1.24	0.538 to 2.85	0.614
Indexed AVA				0.013	0.001 to 0.166	<0.001

AVA, aortic valve area; BMI, body mass index; CI, Confidence interval; EAT, epicardial adipose tissue; HR, Hazard ratio.

**Figure 5:**
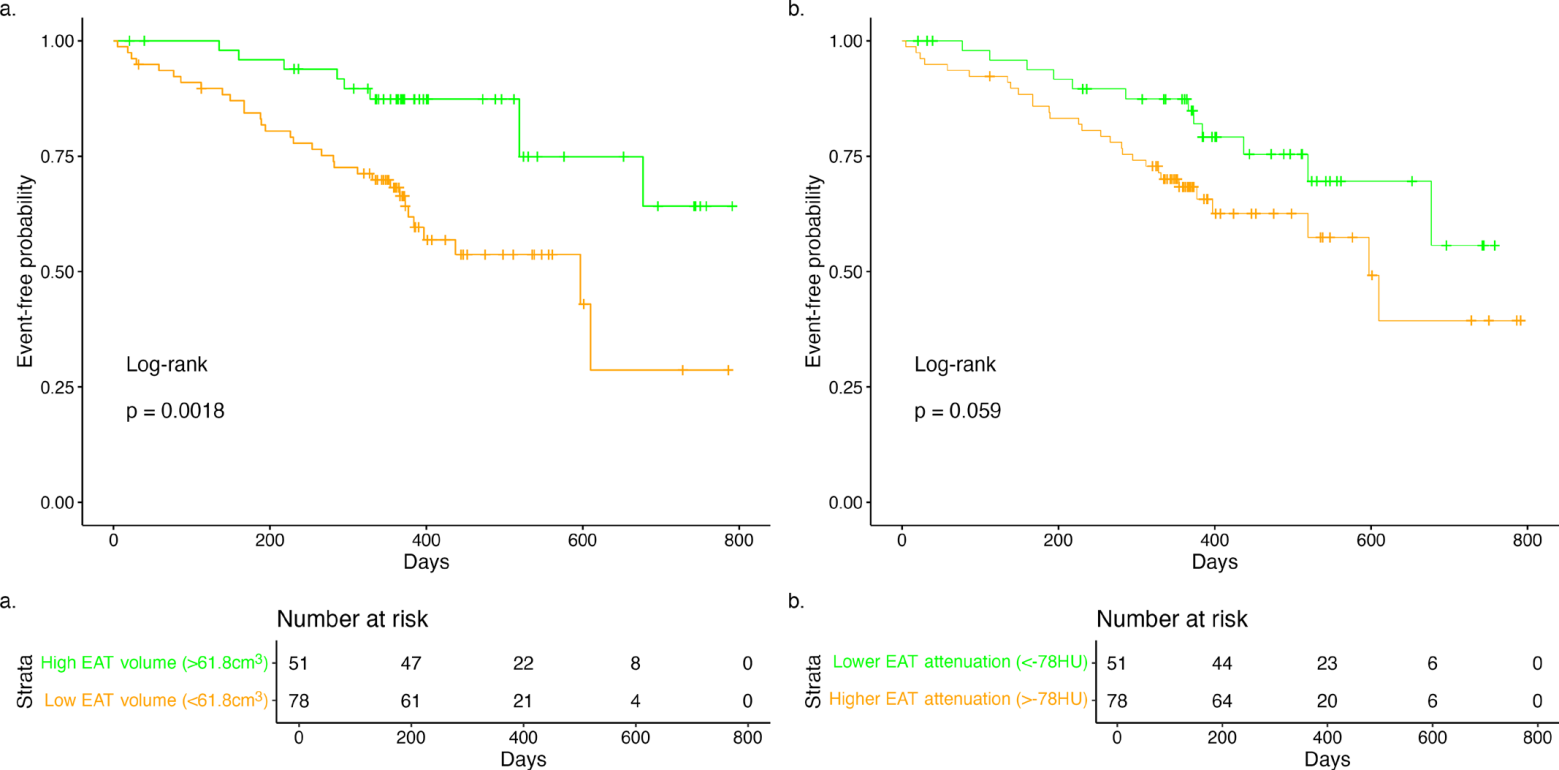
Kaplan-Meier curves for the primary outcome and (a) indexed epicardial adipose tissue (EAT) volume and (b) mean EAT CT attenuation. Median follow-up time, 370 days (IQR 311–473 days) with a censoring proportion of 69%.

The optimal cut-off to define elevated indexed EAT volume was 61.8 cm^3^/m^2^ and for reduced mean CT attenuation was −78 Hounsfield units. Figure 5 shows the Kaplan-Meier curves for both variables using these cut-offs and demonstrates a statistically significantly higher event rate in participants with an indexed EAT volume lower than 61.8 cm^3^/m^2^ compared with a higher indexed EAT volume, with the difference not being statistically significant for EAT attenuation.

## Discussion

In initially asymptomatic patients with moderate-to-severe AS, participants had higher indexed EAT volumes and lower mean CT attenuation, compared with controls. There was a weak positive correlation between EAT volume and AS severity. EAT mean CT attenuation was positively associated with indexed LV mass, but no associations were found between indexed EAT volume and LV remodelling after adjusting for confounding variables. A higher indexed EAT volume was associated with a lower occurrence of symptoms necessitating AVR and cardiovascular events, which was independent of demographic and metabolic factors and AS severity.

### EAT and LV remodelling

Several studies have demonstrated associations between the amount of EAT and LV remodelling, but the assessment of EAT density has been limited. We found a higher mean EAT CT attenuation was associated with an increased indexed LV mass. A higher mean EAT CT attenuation is generally associated with lower volumes of EAT, as seen in this cohort.^9^ Lower EAT CT attenuation may reflect increased triglyceride adipocyte deposition and has been associated with cardiac dysfunction in type 2 diabetes.^16^ A higher mean EAT CT attenuation, however, may be associated with increased inflammation within the deposit due to increased water:lipid ratio, akin to that seen in pericoronary adipose tissue.^22^ Given that in this study a lower mean EAT CT attenuation, which was correlated with a higher EAT volume, was associated with less primary outcome events, the observations seen may reflect a protective role of increased and lower attenuation EAT deposit.

In patients with asymptomatic AS, Arangalage *et al* found CT-derived EAT volume was independently associated with LV mass. However, this study included a wider spectrum of severity of AS, with only 17% meeting the criteria for severe AS.^23^ Additionally, LV mass was measured using echocardiography, whereas the present study used cardiac MRI, which is a more reliable modality for its quantification, and echocardiography often overestimates LV mass.^24^ They also used a wider threshold of Hounsfield units to define adipose tissue from CT. In a similar cohort of patients with at least mild calcific AS, Geers *et al* found indexed EAT volume was correlated with LV mass, global longitudinal strain and septal wall thickness; however, there were no adjustments for any confounding variables.^14^ Furthermore, in severe symptomatic AS, Mancio *et al* demonstrated CT-derived EAT volume was associated with indexed LV mass and LV volumes measured using echocardiography; however, they also did not perform multivariable analyses.^25^ Using echocardiographic measurements, a further study found EAT thickness, a single linear measurement which may not represent the whole epicardial deposit,^9^ was positively correlated with LVMi and they remained associated when adjusted for BMI, diabetes and valve disease severity in patients with severe symptomatic AS.^26^ This study included participants with more progressed disease and had a higher proportion (36%) of patients with diabetes. Patients with diabetes are known to have increased EAT volumes and have previously been shown to have a higher prevalence of concentric LV hypertrophy in AS.^27^ Similar to the current findings, in a cohort of patients with AS of mild-to-moderate severity, CT-derived EAT was not correlated with global longitudinal or circumferential strain from echocardiography, but was weakly correlated with LV end diastolic and systolic pressure.^28^ The present study found indexed EAT volume was associated with circumferential PEDSR, a marker of diastolic function, but this was not independent of age, sex and severity of aortic valve disease.

### EAT, obesity and clinical outcomes in AS

Two previous studies have investigated the association of EAT volume with clinical outcomes in AS. In contradiction to our results, Davin *et al* found that an increased indexed EAT volume measured using MRI was associated with increased cardiovascular death and requirement for symptom-driven surgery, in patients with moderate-to-severe AS.^13^ They adjusted for multiple variables including AVA, relative wall thickness, triglycerides and creatinine but did not adjust for important factors such as sex and BMI. Additionally, the number of asymptomatic participants was small and the follow-up period was longer than the present study at a median of 34 months, with most participants experiencing a clinical outcome during follow-up compared with 31% in this study. Furthermore, the separation seen in event-free survival between participants with high and low EAT volumes occurred after 12 months, which was our median follow-up period. They used MRI to measure EAT volume, which is more technically difficult, as EAT can be harder to differentiate from pericardial adipose tissue and does not provide information on tissue density.^9^ In another study of asymptomatic patients with mild-to-severe AS, with only 17% having severe AS, who were followed up for a median 48 months, Geers *et al* demonstrated that increased EAT volume was associated with all-cause mortality, independent of age, BMI and ejection fraction. They, however, found no correlation between AS severity and EAT. The separation in event-free survival also occurred later than our median follow-up period. This study also explored a different end point compared with ours and the cause of death in the participants was not reported.^14^

Several studies have assessed the role of obesity in the progression of AS. Two previous studies from the same group have identified a lower mortality in patients with obesity compared with patients without obesity, independent of AS severity.^6 7^ However, in another study, which excluded patients with other known cardiovascular disease and diabetes, being overweight or obese was associated with increased total mortality, but not AS-related events, when adjusted for age and other measures.^29^ Additionally, better short-term and long-term outcomes have been reported in patients post transcatheter aortic valve implantation.^8^ Several possible explanations for a possible ‘obesity paradox’ in AS have been proposed, including younger age in patients with obesity with results driven by ‘metabolically healthy’ individuals with obesity, increased cachexia and frailty in patients with low BMIs and increased use of cardioprotective medications in patients with obesity. However, these factors do not explain our novel findings, as most individuals were neither of low nor high body weight, and EAT may exert a separate cardioprotective effect regarding symptom progression in this patient cohort. The mechanism by which this occurs could provide information on the EAT-myocardial interaction in disease states and contribute to the development of EAT as a therapeutic target.

### Strengths and limitations

Participants were prospectively recruited and followed up, with comprehensive phenotyping using multimodality imaging, including CT-derived volumetric and density EAT measurements.^9^ However, this was a post hoc analysis of a previously recruited cohort, and by nature of its observational design, there are likely confounding factors that cannot be overcome by multivariable adjustment with unmeasured confounding factors. CT scans were unavailable for analysis in 20 participants, and EAT measurements could not be obtained in a further 18 scans, contributing to missing data, alongside a small amount of additional missing data. Additionally, interpretation of HRs in assessing event risk should be undertaken with caution given inherent selection bias over time. These factors limit causal inference from the results. Furthermore, all participants were white Caucasian, limiting the generalisability to other populations. Finally, other measures of adiposity aside from BMI were not available, including waist circumference and abdominal visceral adiposity.

## Conclusion

Participants with asymptomatic moderate-to-severe AS have increased EAT volumes and EAT density measured on CT compared with controls. No association was found between indexed EAT volume and markers of LV remodelling in AS, when adjusting for important demographic factors, comorbidities and disease severity. However, lower mean EAT CT attenuation was associated with lower indexed LV mass. Higher indexed EAT volume was associated with a lower occurrence of symptoms necessitating AVR and cardiovascular events. Further studies in larger populations are needed to corroborate these findings and establish whether EAT may have a potentially protective role in symptom progression in AS.

## Supplementary material

10.1136/openhrt-2025-003804 online supplemental file 1

## Data Availability

Data are available on reasonable request.
