# Supplementary material for: Association of epicardial adipose tissue with markers of cardiac remodelling and clinical outcomes in asymptomatic aortic stenosis
Source: Open Heart. 2026 Feb 9;13(1):e003804. doi: 10.1136/openhrt-2025-003804 (PMC12887484; doi:10.1136/openhrt-2025-003804)
Supplement: online supplemental file 1 [file openhrt-13-1-s001.docx]

Supplementary Tables and Figures

| Supplementary Table 1: Echocardiography results in participants with aortic stenosis | | |
| --- | --- | --- |
|  | **Aortic Stenosis**  n = 136 | |
| AS severity - severe | 96 (71%) | |
| Aortic valve area (cm^2^) | 1.1 (0.9, 1.4) | |
| Peak pressure gradient (mmHg) | 54.5 (47.9, 70.5) | |
| Mean pressure gradient (mmHg) | 32.3 (26.0, 40.5) | |
| Aortic valve peak flow (m/s) | 3.7 (3.5, 4.2) | |
| AR severity – Moderate or Moderate/Severe | 5 (3.7%) | |
| MR severity - Mild or Mild/Moderate | 44 (32%) | |
| E wave (cm/sec) | 73.6 (61.2, 89.9) | |
| A wave (cm/sec) | 89.1±21.7 | |
| E to A ratio | 0.8 (0.7, 1.0) | |
| Septal e' (cm/sec) | 5.6 (4.6, 6.8) | |
| Lateral e' (cm/sec) | 7.7 (5.5, 9.7) | |
| Septal E/e' | 12.7 (10.5, 15.4) | |
| Lateral E/e' | 10.0 (7.5, 12.1) | |
| Mean ± SD, Median (IQR) or n (%)  AR, aortic regurgitation; AS, aortic stenosis; MR, mitral regurgitation | |  |


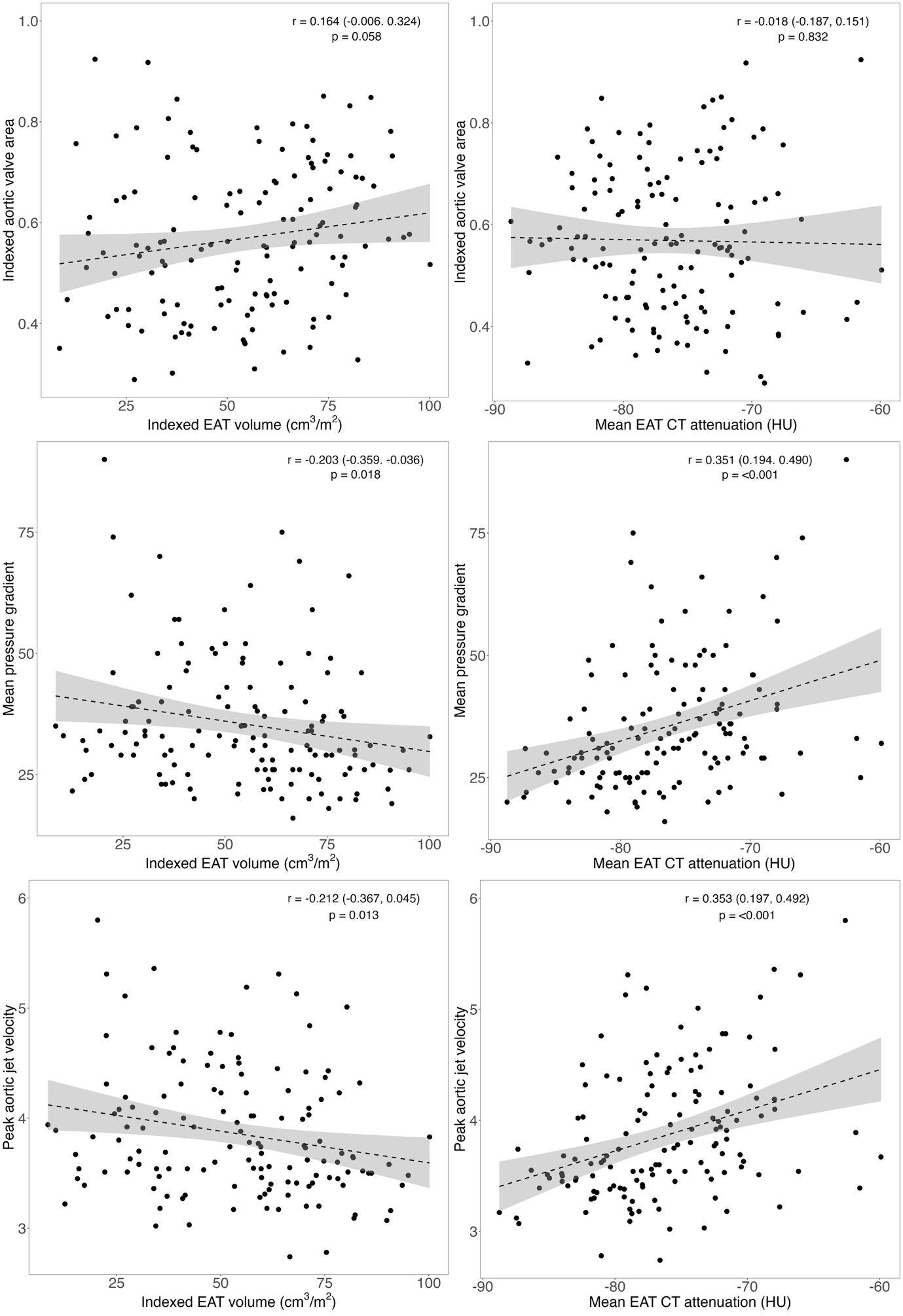


Supplementary Figure 1: The association of indexed EAT volume and mean EAT CT attenuation with measures of aortic stenosis severity

CT, computed tomography; EAT; epicardial adipose tissue

Supplementary Table 2: Multivariable regression for EAT and cardiac MRI measures

|  | **Beta co-efficients (95% CI)** | **p-value** | **Beta co-efficients (95% CI)** | **p-value** | **Beta co-efficients (95% CI)** | **p-value** |
| --- | --- | --- | --- | --- | --- | --- |
| **LV mass/volume ratio** |  |  |  |  |  |  |
| Age (years) | 0.00162 (-0.00116, 0.00439) | 0.25 | 0.00104 (-0.00204, 0.00411) | 0.51 | 0.00149 (-0.00144, 0.00442) | 0.32 |
| Sex (Male) | 0.0718 (-0.0101, 0.154) | 0.085 | 0.0712 (-0.0108, 0.153) | 0.088 | 0.0722 (-0.01, 0.154) | 0.085 |
| BMI (kg/m^2^) | 0.00431 (-0.00507, 0.0137) | 0.36 | 0.00299 (-0.00687, 0.0129) | 0.55 | 0.00374 (-0.00653, 0.014) | 0.47 |
| Systolic blood pressure (mmHg) | -0.00124 (-0.00303, 0.00056) | 0.17 | -0.00119 (-0.00299, 0.00061) | 0.19 | -0.0012 (-0.00302, 0.00061) | 0.19 |
| Diabetes status | 0.0456 (-0.0613, 0.153) | 0.4 | 0.0429 (-0.0643, 0.15) | 0.43 | 0.0458 (-0.0616, 0.153) | 0.4 |
| Indexed AVA (cm^2^/m^2^) | -0.399 (-0.655, -0.144) | 0.002 | -0.431 (-0.697, -0.166) | 0.002 | -0.402 (-0.66, -0.145) | 0.002 |
| Indexed total EAT volume (cm^3^/m^2^) |  |  | 0.00089 (-0.00113, 0.00291) | 0.39 |  |  |
| Mean EAT CT attenuation (HU) |  |  |  |  | -0.00103 (-0.00841, 0.00635) | 0.78 |
|  | Adjusted R^2^ = 0.084 | | Adjusted R^2^ = 0.082 | | Adjusted R^2^ = 0.077 | |
|  | p = 0.009 | | p = 0.013 | | p = 0.016 | |
| **Indexed LV mass** |  |  |  |  |  |  |
| Age (years) | -0.358 (-0.563, -0.153) | <0.001 | -0.299 (-0.526, -0.0716) | 0.01 | -0.259 (-0.468, -0.0494) | 0.016 |
| Sex (Male) | 11.2 (5.15, 17.3) | <0.001 | 11.3 (5.22, 17.3) | <0.001 | 10.9 (5.02, 16.8) | <0.001 |
| BMI (kg/m^2^) | 0.389 (-0.305, 1.08) | 0.27 | 0.524 (-0.204, 1.25) | 0.16 | 0.839 (0.105, 1.57) | 0.025 |
| Systolic blood pressure (mmHg) | 0.111 (-0.0214, 0.244) | 0.1 | 0.106 (-0.0262, 0.239) | 0.11 | 0.0844 (-0.0454, 0.214) | 0.2 |
| Diabetes status | -1.88 (-9.79, 6.03) | 0.64 | -1.6 (-9.52, 6.31) | 0.69 | -2 (-9.66, 5.67) | 0.61 |
| Indexed AVA (cm^2^/m^2^) | -5.48 (-24.4, 13.4) | 0.57 | -2.21 (-21.8, 17.4) | 0.82 | -3.12 (-21.5, 15.3) | 0.74 |
| Indexed total EAT volume (cm^3^/m^2^) |  |  | -0.0903 (-0.239, 0.0587) | 0.23 |  |  |
| Mean EAT CT attenuation (HU) |  |  |  |  | 0.808 (0.281, 1.33) | 0.003 |
|  | Adjusted R^2^ = 0.126 | | Adjusted R^2^ = 0.129 | | Adjusted R^2^ = 0.179 | |
|  | p < 0.001 | | p < 0.001 | | p < 0.001 | |
| **GLS** |  |  |  |  |  |  |
| Age (years) | 0.0118 (-0.0216, 0.0451) | 0.49 | 0.0159 (-0.0212, 0.0529) | 0.4 | 0.0116 (-0.0235, 0.0468) | 0.51 |
| Sex (Male) | -1.4 (-2.39, -0.412) | 0.006 | -1.39 (-2.38, -0.402) | 0.006 | -1.4 (-2.39, -0.408) | 0.006 |
| BMI (kg/m^2^) | -0.0929 (-0.206, 0.0203) | 0.11 | -0.0835 (-0.203, 0.0358) | 0.17 | -0.0934 (-0.218, 0.0309) | 0.14 |
| Systolic blood pressure (mmHg) | -0.0283 (-0.05, -0.00661) | 0.011 | -0.0285 (-0.0503, -0.00673) | 0.011 | -0.0283 (-0.0502, -0.00633) | 0.012 |
| Diabetes status | 0.917 (-0.398, 2.23) | 0.17 | 0.931 (-0.389, 2.25) | 0.17 | 0.917 (-0.404, 2.24) | 0.17 |
| Indexed AVA (cm^2^/m^2^) | 2.69 (-0.421, 5.8) | 0.089 | 2.91 (-0.324, 6.14) | 0.077 | 2.69 (-0.441, 5.82) | 0.092 |
| Indexed total EAT volume (cm^3^/m^2^) |  |  | -0.00632 (-0.0308, 0.0182) | 0.61 |  |  |
| Mean EAT CT attenuation (HU) |  |  |  |  | -0.0009 (-0.0908, 0.089) | 0.98 |
|  | Adjusted R^2^ = 0.112 | | Adjusted R^2^ = 0.107 | | Adjusted R^2^ = 0.105 | |
|  | p = 0.002 | | p = 0.004 | | p = 0.004 | |
| **Circumferential PEDSR** |  |  |  |  |  |  |
| Age (years) | -0.00717 (-0.00998, -0.00437) | <0.001 | -0.00721 (-0.0103, -0.00411) | <0.001 | -0.00732 (-0.0103, -0.00438) | <0.001 |
| Sex (Male) | -0.136 (-0.219, -0.0537) | 0.001 | -0.136 (-0.219, -0.0534) | 0.001 | -0.136 (-0.219, -0.0527) | 0.002 |
| BMI (kg/m^2^) | -0.00861 (-0.018, 0.0008) | 0.072 | -0.00871 (-0.0187, 0.00124) | 0.086 | -0.00932 (-0.0196, 0.00098) | 0.076 |
| Systolic blood pressure (mmHg) | -0.00031 (-0.00213, 0.00151) | 0.74 | -0.00031 (-0.00214, 0.00153) | 0.74 | -0.00027 (-0.00211, 0.00158) | 0.77 |
| Diabetes status | 0.0375 (-0.0746, 0.15) | 0.51 | 0.0373 (-0.0754, 0.15) | 0.51 | 0.0376 (-0.075, 0.15) | 0.51 |
| Indexed AVA (cm^2^/m^2^) | 0.137 (-0.121, 0.395) | 0.29 | 0.135 (-0.134, 0.404) | 0.32 | 0.134 (-0.125, 0.394) | 0.31 |
| Indexed total EAT volume (cm^3^/m^2^) |  |  | 0.00007 (-0.00201, 0.00215) | 0.95 |  |  |
| Mean EAT CT attenuation (HU) |  |  |  |  | -0.0013 (-0.00882, 0.00622) | 0.73 |
|  | Adjusted R^2^ = 0.293 | | Adjusted R^2^ = 0.287 | | Adjusted R^2^ = 0.288 | |
|  | p < 0.001 | | p < 0.001 | | p < 0.001 | |
| **MPR** |  |  |  |  |  |  |
| Age (years) | -0.0184 (-0.0278, -0.00894) | <0.001 | -0.0171 (-0.0274, -0.00681) | 0.001 | -0.0199 (-0.0297, -0.01) | <0.001 |
| Sex (Male) | 0.144 (-0.132, 0.419) | 0.3 | 0.144 (-0.132, 0.42) | 0.3 | 0.15 (-0.126, 0.426) | 0.28 |
| BMI (kg/m^2^) | -0.00268 (-0.034, 0.0286) | 0.87 | 0.00092 (-0.0326, 0.0344) | 0.96 | -0.0105 (-0.0452, 0.0242) | 0.55 |
| Systolic blood pressure (mmHg) | 0.00121 (-0.0048, 0.00721) | 0.69 | 0.00116 (-0.00486, 0.00718) | 0.7 | 0.00152 (-0.00451, 0.00755) | 0.62 |
| Diabetes status | -0.32 (-0.671, 0.0314) | 0.074 | -0.314 (-0.666, 0.0389) | 0.081 | -0.318 (-0.669, 0.0327) | 0.075 |
| Indexed AVA (cm^2^/m^2^) | 0.759 (-0.0875, 1.6) | 0.078 | 0.832 (-0.0488, 1.71) | 0.064 | 0.733 (-0.115, 1.58) | 0.09 |
| Indexed total EAT volume (cm^3^/m^2^) |  |  | -0.00207 (-0.00873, 0.0046) | 0.54 |  |  |
| Mean EAT CT attenuation (HU) |  |  |  |  | -0.0128 (-0.0371, 0.0116) | 0.3 |
|  | Adjusted R^2^ = 0.141 | | Adjusted R^2^ = 0.136 | | Adjusted R^2^ = 0.141 | |
|  | p < 0.001 | | p < 0.001 | | p < 0.001 | |
| Between 2 and 10 missing data points were missing for linear regression models with 12% of participants having at least one variable missing.  AVA, Aortic valve area; BMI, body mass index; CI. confidence intervals; CT, computed tomography; EAT, epicardial adipose tissue; GLS, global longitudinal strain; HU, Hounsfield units; LV, left ventricular; MPR, myocardial perfusion reserve; PEDSR, peak early diastolic strain rate | | | | | | |

|  |
| --- |
